# A review of protocols for the experimental release of kelp (Laminariales) zoospores

**DOI:** 10.1002/ece3.5389

**Published:** 2019-06-20

**Authors:** Nahlah A. Alsuwaiyan, Margaret B. Mohring, Marion Cambridge, Melinda A. Coleman, Gary A. Kendrick, Thomas Wernberg

**Affiliations:** ^1^ School of Biological Sciences and UWA Oceans Institute University of Western Australia Crawley Western Australia Australia; ^2^ Department of Biology Unaizah College of Sciences and Arts, Qassim University Unaizah Saudi Arabia; ^3^ Department of Parks and Wildlife Kensington Western Australia Australia; ^4^ National Marine Science Centre Southern Cross University Coffs Harbour New South Wales Australia; ^5^ Department of Primary Industries National Marine Science Centre Coffs Harbour New South Wales Australia

**Keywords:** desiccation, gametophytes, kelp forests, Laminariales, zoospore release, zoospores

## Abstract

**Abstract:**

Kelps (order Laminariales) are foundation species in temperate and arctic seas globally, but they are in decline in many places. Laminarian kelp have an alternation of generations and this poses challenges for experimental studies due to the difficulties in achieving zoospore release and gametophyte growth. Here, we review and synthesize the protocols that have been used to induce zoospore release in kelps to identify commonalities and provide guidance on best practices. We found 171 papers, where zoospore release was induced in four kelp families from 35 different ecoregions. The most commonly treated family was Laminariaceae, followed by Lessoniaceae and the most studied ecoregion was Central Chile, followed by the Southern California Bight. Zoospore release generally involved three steps: a pretreatment which included cleaning of the reproductive tissue to eliminate epiphytic organisms, followed by desiccation of the tissue, and finally a postdesiccation immersion of the reproductive material in a seawater medium for zoospore release. Despite these commonalities, there was a high degree of variation in the detail within each of these steps, even among studies within genera and from the same ecoregions. This suggests either that zoospore release may be relatively insensitive across the Laminariales or that little methods optimization has been undertaken. We suggest that greater attention to standardization of protocols and reporting of methodology and optimization would improve comparisons of kelp zoospore release across species and locations and facilitate a broader understanding of this key, but understudied life history stage.

**Open Research Badges:**

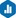

This article has earned an Open Data Badge for making publicly available the digitally‐shareable data necessary to reproduce the reported results. The data is available at https://doi.org/10.5061/dryad.0kh1f8j.

## 1 INTRODUCTION

Kelp forests are among the most diverse and productive ecosystems in temperate and polar seas worldwide (Mann, 1973; Steneck et al., 2002; Wernberg, Krumhansl, Filbee‐Dexter, & Pedersen, 2019). They provide shelter and habitat for many marine animals (Teagle, Hawkins, Moore, & Smale, 2017), and are an important food source for many organisms within the kelp forests and neighboring ecosystems (Dayton, 1985; Krumhansl & Scheibling, 2012). Kelp forests form the base of the food chain for many commercially important species, such as abalone, sea urchins, crab, lobster, and fish (Bennett et al., 2016; Bertocci, Araújo, Oliveira, & Sousa‐Pinto, 2015; Britton‐Simmons et al., 2012; Dayton, Tegner, Edwards, & Riser, 1998; Kelly, Krumhansl, & Scheibling, 2012; Steneck et al., 2002). They also play an important role in marine biogeochemical cycles through storing and regulating carbon and nitrogen (Duarte, Losada, Hendriks, Mazarrasa, & Marba, 2013; Smith, 1981).

Kelp forests are under increasing threat by a range of anthropogenic stressors including kelp harvesting (Christie, Fredriksen, & Rinde, 1998; Lorentsen, Sjotun, & Gremillet, 2010), overfishing (Tegner & Dayton, 2000), overgrazing by range‐extending species such as sea urchins and fishes (Bennett, Wernberg, Harvey, Santana‐Garcon, & Saunders, 2015; Ling, Johnson, Ridgway, Hobday, & Haddon, 2009; Vergés et al., 2014), increasing seawater temperatures (Filbee‐Dexter, Feehan, & Scheibling, 2016; Müller, Laepplea, Bartsch, & Wiencke, 2009; Raybaud et al., 2013; Wernberg et al., 2016), and storms (Filbee‐Dexter & Scheibling, 2012; Reed et al., 2011), and decreased water quality (Airoldi, 2003; Connell et al., 2008; Delebecq et al., 2013; Strain, Thomson, Micheli, Mancuso, & Airoldi, 2014). These stressors are likely to have far‐reaching implications for kelp forests resulting in shifts from diverse, three‐dimensional kelp forests to structurally poor and depauperate turf‐dominated communities (Filbee‐Dexter & Wernberg, 2018). Indeed, a recent global analysis of kelp forest time series >20 years found that 61% of the world's kelp forests have been in decline over the past five decades as a result of one or more of the above mechanisms (Krumhansl et al., 2016; Wernberg et al., 2019). Because kelp forests are foundation species, their loss or displacement by other species has serious consequences, affecting biodiversity, ecological function, biogeochemical cycling, and human communities.

Kelps have a heteromorphic diplohaplontic life cycle, with two morphologically distinct life stages: microscopic haploid gametophytes and macroscopic diploid sporophytes. Compared to the intensely studied macroscopic sporophytes, substantially fewer studies have examined the microscopic gametophyte and early sporophyte stages, yet these parts of the kelp life cycle have been identified as bottlenecks in our current understanding of the ecology of kelp populations (Schiel & Foster, 2006). Research into the microscopic phases of the kelp life cycle has been firmly centered around the survival and growth of gametophytes and juvenile sporophytes, including the effects of varied light and temperature conditions (Bolton & Levitt, 1985; Fejtek, Edwards, & Kim, 2011; Mohring, Wernberg, Wright, Connell, & Russell, 2014; Novaczek, 1984b; Tatsumi & Wright, 2016), and the influence of sediments, nutrients, and toxic contaminants (Amsler & Neushul, 1990; Bidwell, Wheeler, & Burridge, 1998; Devinny & Volse, 1978). Other studies have quantified zoospore release density (Mohring, Wernberg, Kendrick, & Rule, 2013; Reed, Anderson, Ebeling, & Anghera, 1997), zoospore swimming capability (Amsler & Neushul, 1989), settlement and recruitment success (Reed, 1990), and the effects of different settlement densities on survival and growth of gametophytes (Choi, Kim, Lee, Park, & Nam, 2005). Importantly, almost all of these studies have been done in the laboratory and we have little understanding of how such processes translate into natural settings.

The relative scarcity of studies on microscopic gametophytes and sporophytes is, at least in part, due to methodological challenges, since almost any work on the microscopic stages of kelps requires the release of a high volume of healthy, viable kelp zoospores for subsequent experimentation. Throughout the global distribution of kelp and across taxa, different protocols have been used to achieve zoospore release. There are only few studies that examined kelp zoospore release in the field (e.g., Anderson & North, 1966; Joska & Bolton, 1987), and our understanding of what cues release is scant relative to other well studied seaweed taxa (e.g., fucoids, Pearson & Serrão, 2006). Thus, translating knowledge of natural cues into laboratory settings to induce zoospore release has not been possible to date. This review aims to identify and synthesize successful methodologies that have been used to induce zoospore release in order to provide guidance on best practices. In doing so, we hope to promote optimization and a more unified approach, and increase the comparability across studies on zoospores and microscopic stages of kelps.

## 2 METHODS

Here, we focused on Laminarian kelp (species within the order Laminariales). We searched the ISI databases (Web of Science, Current Contents, and One Search university catalogue) for peer‐reviewed papers using various combinations of “kelp,” “Laminariales,” “spores,” “gametophytes,” and “sporulation.” All papers returned were examined and further literature was found by back‐tracking from their reference lists. We stopped searching on February 15, 2018, to allow a definitive analysis. Overall, we located 171 papers where kelp zoospore release had been induced in the laboratory. From each paper, we extracted information on the species studied, geographic location (GPS position) and ecoregion, collection date, sea surface temperature, aim of the research, and details concerning the protocol used to induce zoospore release. Details on all papers and data extracted are freely available on Dryad (see data availability statement). Where position was not reported in the study, approximate GPS coordinates were estimated using Google Earth. Studies were assigned to ecoregions, provinces, and realms corresponding to Spalding et al. (2007)'s “Marine Ecoregions of the World.” Where sufficient information was available (i.e., time of collection and geographic location), monthly NOAA Optimum Interpolation (OI) sea surface temperature (SST) version 2 dataset (downloaded from http://www.esrl.noaa.gov/psd/) was used to obtain SST data. This product contains data from December 1981 to present. Many papers reported on multiple species, and in this synthesis we treat each genus as a separate study. All nomenclature was updated to report most recent names according to AlgaeBase (Guiry & Guiry, 2018). In the analyses, where time was expressed as “overnight,” a 12‐hr period was designated. Also, to be consistent, the terms “wiped,” “blotted,” and “dried” were grouped under the term wiped, and “washed” and “rinsed” were grouped under rinsed.

## 3 RESULTS

### 3.1 Research questions, taxa and regions

**Figure 1:**
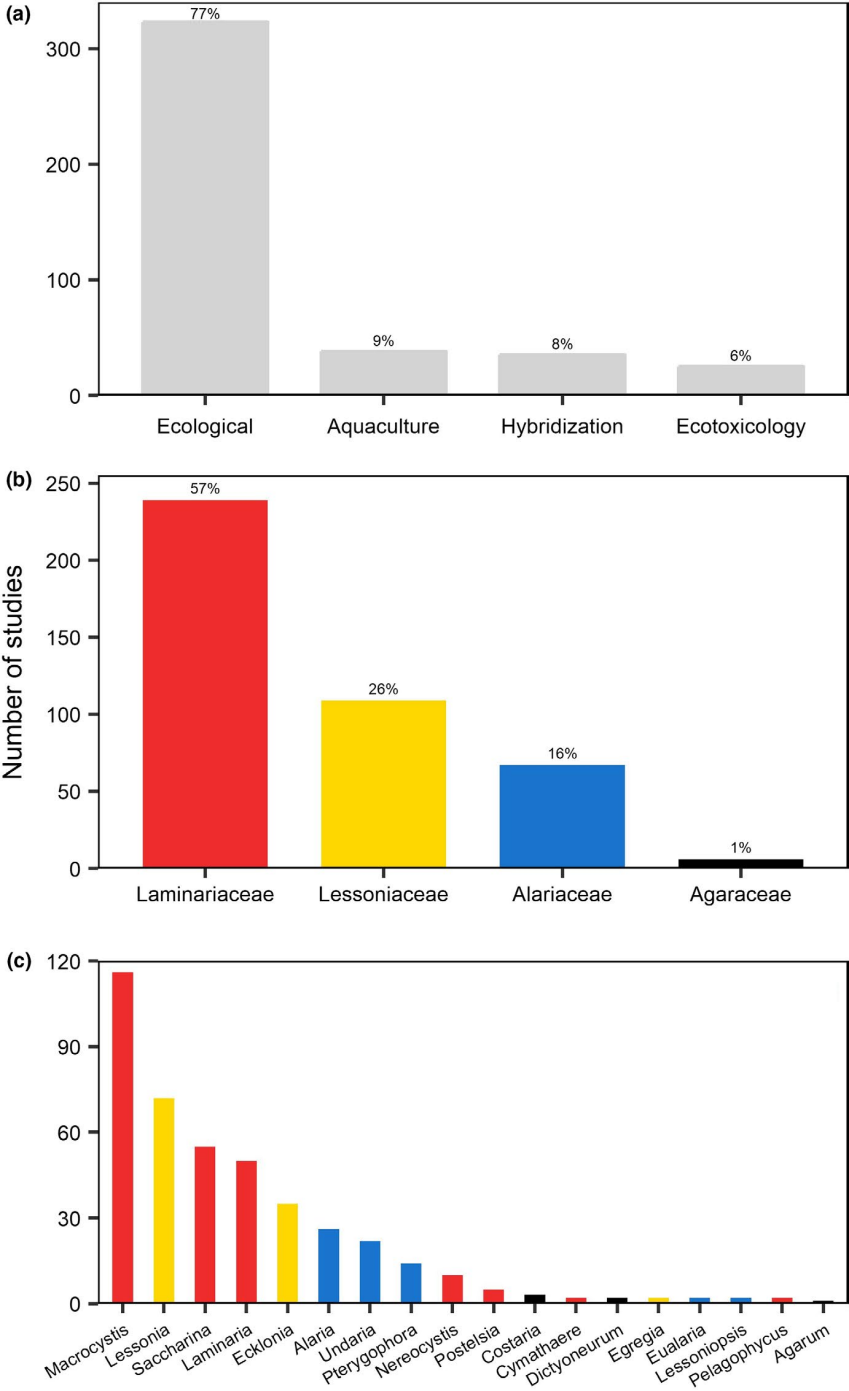
Characteristics of artificial zoospore extraction studies (171 papers, 421 studies). Classification of studies according to (a) the research question addressed; (b) the family studied; and (c) the genera studied. All papers and their classifications can be found in the raw data table provided on Dryad; see data availability statement

A total of 421 studies were extracted from the 171 papers (raw data provided on Dryad; see data availability statement). The vast majority (77%) were ecological studies evaluating growth, survival, and mortality of zoospores, gametophytes, or microscopic sporophytes under different experimental conditions (Figure 1a). The remaining studies included aquaculture (9%), hybridization (8%), and ecotoxicology (6%) studies.

The 421 zoospore release studies encompassed four families and 18 of the 33 existing kelp genera (Bolton, 2010) (Figure 1b,c). The most commonly studied family was the Laminariaceae (57%), where half (116 studies) were on the genus *Macrocystis*. Furthermore, 26% of the studies were on species from the family Lessoniaceae, including *Lessonia* spp. (72 studies), *Ecklonia* spp. (35), and *Egregia* spp. (2). Only 1% (6 studies) out of the total studies were on species from the Agaraceae family.

**Figure 2:**
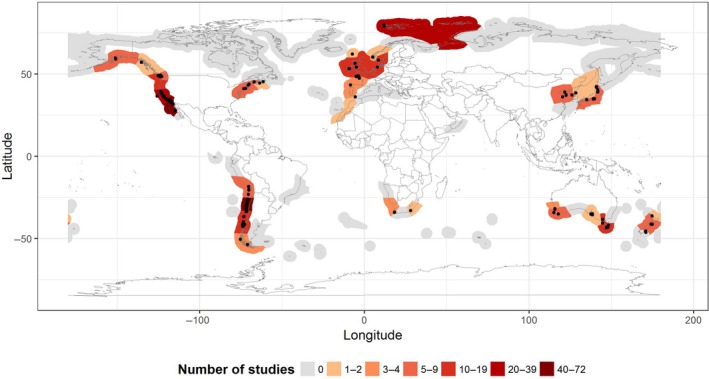
Global distribution of zoospore extraction based experiments. Ecoregions are colored based on the number of studies conducted and gray shading indicates ecoregions where kelps are present but for which no data were available. Black dots show the exact geographical location of the sites used for the collection of kelp tissue

Nine papers were excluded from the evaluation of geographical patterns in the zoospore release studies, as GPS information could not be identified (cf raw data provided on Dryad; see data availability statement). The remaining 162 papers represented studies from 35 of the 99 global ecoregions where kelps exist (Krumhansl et al., 2016) (Figure 2), and were, not surprisingly, dominated by temperate ecoregions (91%). The remaining studies were on species from the Arctic, and were focused in two locations in the western side of the North and East Barents Sea. The highest number of studies were conducted in Central Chile (18%), followed by the Southern California Bight (17%). Of all studied ecoregions, 69% had less than 10 studies each.

### 3.2 Zoospore release protocols: pretreatment, desiccation, and immersion

There was a high degree of variation across studies in the protocols used to induce zoospore release and rarely was there biological justification for protocols. Nevertheless, zoospore release generally involved three major steps: (a) a pretreatment involving brushing, washing, and cleaning of the reproductive tissue to eliminate epiphytic diatoms, (b) desiccation of the tissue for a period of time, and (c) a postdesiccation immersion of the reproductive material in a seawater medium for final zoospore release.

**Figure 3:**
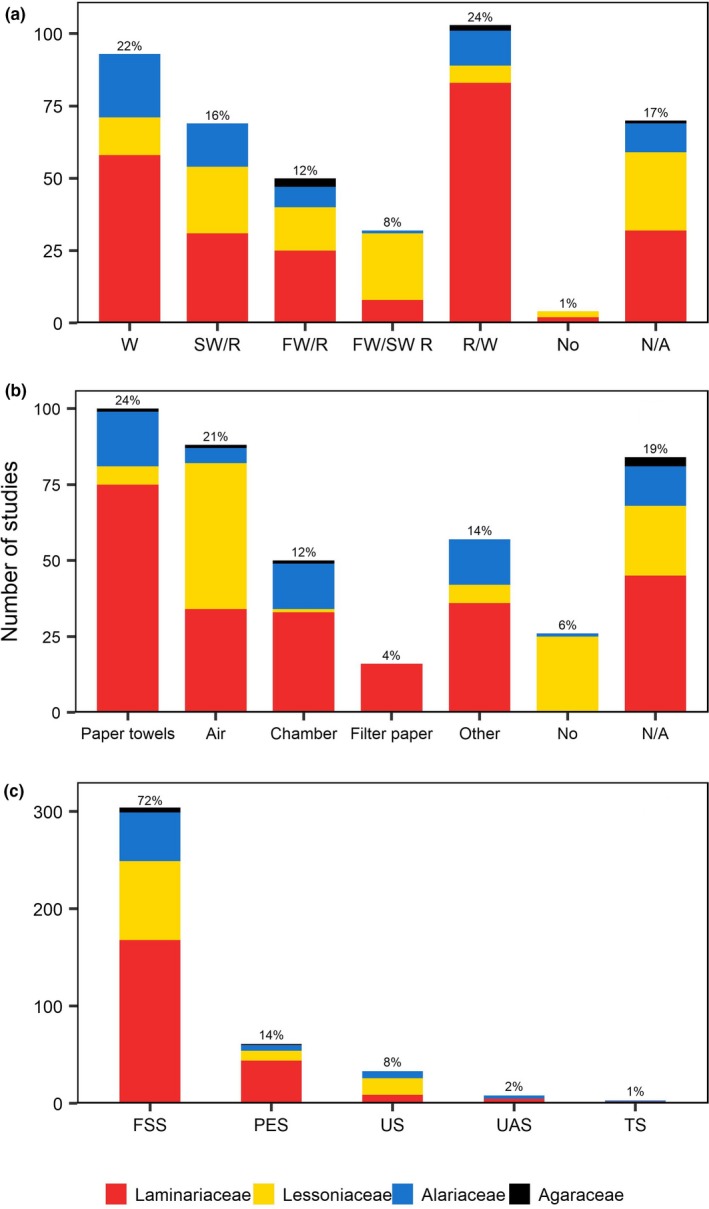
Zoospore release protocol: (a) pretreatment method where W, wiped clean and dried; SW/R, seawater rinse; FW/R, fresh water rinse; FW/SW R, fresh water and seawater rinse; R/W, rinsed and wiped; No, no pretreatment; N/A, no data available; (b) desiccation environments where No, no desiccation treatment; N/A, no data available; (c) postdesiccation immersion media where FSS, filtered‐sterilized seawater; PES, Provasoli‐enriched seawater; US, unfiltered seawater; UAS, unenriched artificial seawater; TS, Tyndallized seawater. Colors refer to the families studied

A range of pretreatment protocols were reported (Figure 3a). Many studies wiped clean the reproductive tissue with cotton towels, paper towels, or tissue paper (22%), while some rinsed it in seawater (16%), fresh water (12%), or a combination of both (8%). Moreover, 24% of the studies used a combination of wiping and rinsing of the reproductive material. Only 1% of the studies explicitly reported no pretreatment of the reproductive tissue; however, 17% either did not pretreat the tissue, or did not include this information in their methods. In some instances, chemicals such as iodine, bleach, chlorine, and ethanol were used during the pretreatment process (9%, 39 studies). Two studies exposed the fertile tissue to ultrasound for 20 s before rinsing with filtered seawater.

**Figure 4:**
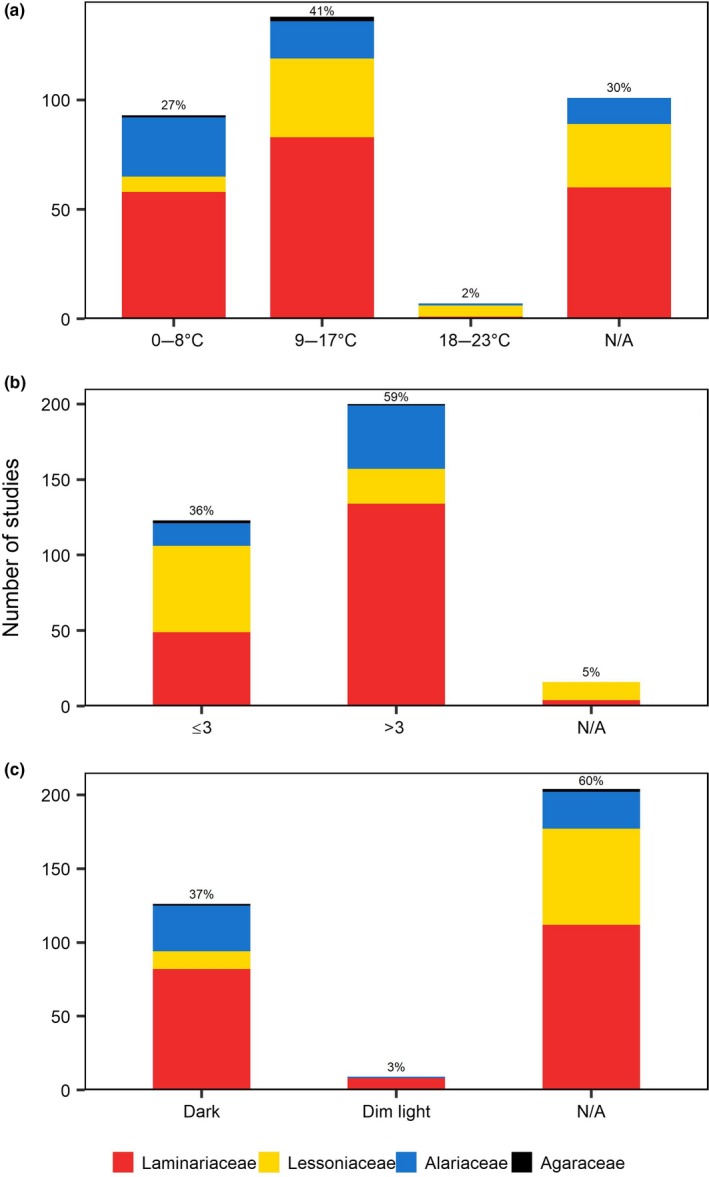
Zoospore release desiccation treatment: (a) temperature; (b) period (hr); (c) light conditions. Colors refer to the families studied

After pretreatment, most studies (81%) desiccated the reproductive tissue in order to induce kelp zoospore release. A range of different drying environments were used during desiccation (Figure 3b) but the most common were wrapping in moist paper towels (24%), followed by air drying (21%). The temperature at which the reproductive material was desiccated also varied (Figure 4a). For individual studies, desiccation temperatures ranged from 0°C to 20–23°C. The most commonly used desiccation temperatures were between 9°C and 17°C (41%). Moreover, 30% of the studies either did not state the desiccation temperature used or included the term “room temperature” without specifying the actual temperature at which thalli were allowed to air‐dry.

Desiccation times ranged from less than 15 min to up to 48 hr. More than half (59%) of the total studies desiccated the tissue for >3 hr (Figure 4b) and the most common period used was 12 hr (38%). For studies that desiccated the tissue for less than 3 hr, 1 hr was the most common period (19%). Approximately 37% of the studies desiccated the reproductive tissues in total darkness but the majority (60%) did not specify light conditions during desiccation (Figure 4c).

**Figure 5:**
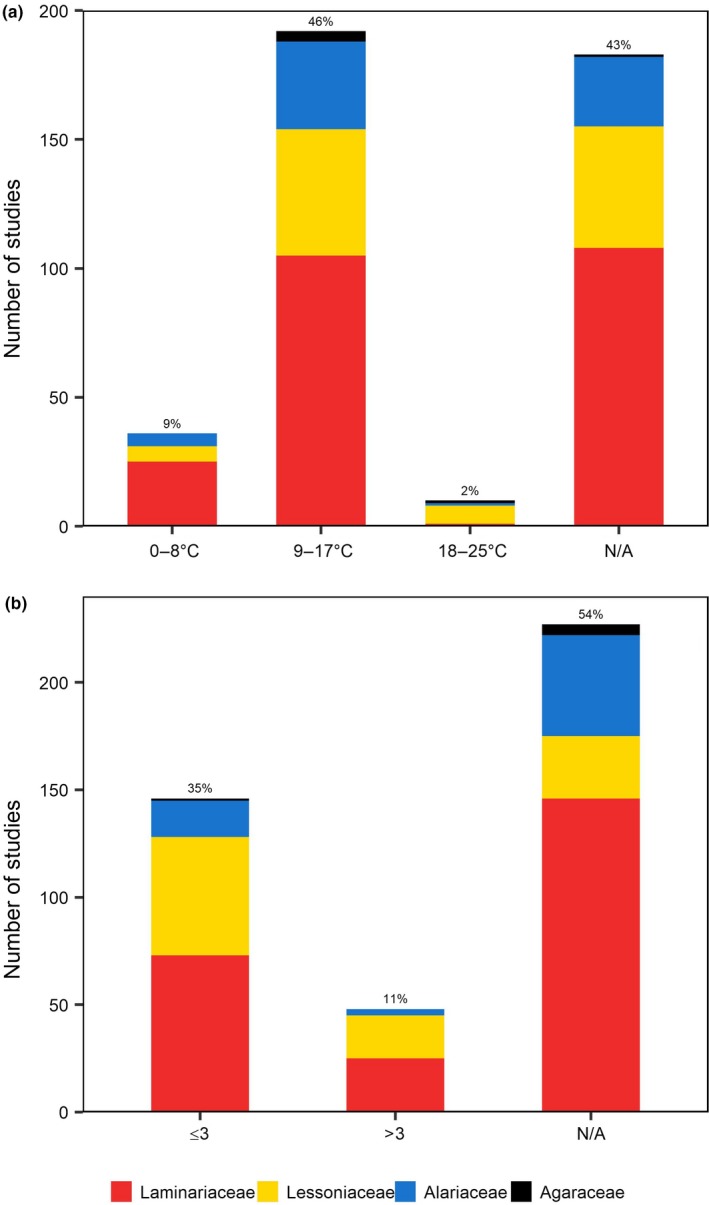
Zoospore release postdesiccation immersion: (a) temperature; (b) period (hr). Colors refer to the families studied

Once drying time had elapsed, the reproductive tissues were submerged in a variety of culture media (Figure 3c); including filtered‐sterilized (72%), Provasoli‐enriched (14%), unfiltered (8%), unenriched artificial (2%), and Tyndallized (1%) seawater. Three studies (1%) agitated the fertile tissue in a copper medium and four studies (1%) added the tissue to a modified f/2 culture medium. Temperatures during submergence ranged from 0°C to 25°C, with the most commonly used temperatures between 9°C and 17°C (46%, Figure 5a).

Furthermore, 54% of the studies did not report the postdesiccation period used, whereas 35% immersed the tissue for a short period of time ≤3 hr (Figure 5b), most commonly 1 hr (16%). Only 27% specified the light conditions during the postdesiccation period, and conditions ranged from ambient light (7%) to total darkness (8%).

Correlations between desiccation/immersion temperatures and ambient seawater temperature were possible to perform on 56% of the total studies. The remaining studies lacked sufficient information (i.e., time of collection or geographic location). Of all studies, 24% desiccated the tissue at temperatures similar to what they would have been subjected to in the field, and 20% submerged the tissue at temperatures similar to ambient seawater temperature in the region. Only 5% selected both desiccation and immersion temperatures to represent in situ sea temperatures. The remaining 51% either desiccated/immersed the tissue at temperatures not representative of ambient seawater temperature, did not report both temperatures used, or used the term room temperature without specifying the actual temperature.

A total of 166 studies (39%) adopted their methods from previous studies with minor modifications. Only one study mentioned any prior assessment of their zoospore release protocol in terms of consequences for quantity or health of the released zoospores.

## 4 DISCUSSION

Knowledge of the processes that influence the gametophyte and microscopic sporophyte stages of kelp is vital to understanding and predicting kelp responses to changing environmental conditions, but is hindered by the challenges involved in studying the early life stages. Zoospore release is a critical first step in the life cycle of kelps, and a prerequisite for culturing and studying their microscopic life stages. Our literature review revealed over 421 studies that utilized zoospore release protocols, with a strong bias toward ecological experiments testing zoospore and gametophyte performance under a range of environmental conditions (cf raw data provided on Dryad; see data availability statement) including temperature (Mabin, Gribben, Fischer, & Wright, 2013; Matson & Edwards, 2007; Mohring et al., 2014), UV radiation (Han & Kain, 1992; Huovinen, Oikari, Soimasuo, & Cherr, 2000), and sedimentation (Deiman, Iken, & Konar, 2012; Geange, Powell, Clemens‐Seely, & Cárdenas, 2014). This focus can likely be attributed to an interest in understanding the growing impacts of human activities on kelp forests. With such a burgeoning interest and pressing need for information on kelp response to environmental change, standardizing and optimizing methodologies so that studies are comparable and not influenced by methodological artifacts, would bring strong benefits in terms of synthesizing and interpreting the collective knowledge.

Despite the abundance of studies that reported zoospore release protocols, there were only broad commonalities among methodologies with some general similarities in the sequence of events used to induce zoospore release. Most studies generally followed the same methodological framework with (a) pretreatment to remove epiphytes, (b) a period of desiccation, and (c) submergence in seawater or media. This general protocol is likely reflective of either the biological basis for zoospore release in nature or based on methodology for other taxa. For example, fucoid algae have a long history of experimental life history study and techniques for gamete release have a strong biological basis (see review by Pearson & Serrão, 2006) that are somewhat similar to the general protocols for kelps. Relative to fucoids with their simple life histories, there are substantially greater difficulties involved in measuring kelp zoospore release and subsequent gametophyte processes in situ. A study that examined kelp zoospore release in the field found that zoospore extraction can be induced in the laboratory, but at the same time release will not occur naturally in situ (Joska & Bolton, 1987). This likely means that experimental zoospore release protocols are more reflective of trial and error than a sound biological basis. It is perhaps not surprising then that only one study (McConnico & Foster, 2005) optimized methodology and provided an explicit rationale for their choice of protocol. In this study, treatments were developed through testing to optimize the abundance of the released zoospores (McConnico & Foster, 2005). This study demonstrates that choice of zoospore release protocol can have substantial impacts on the abundance or subsequent viability of zoospores and their performance in experimental treatments, and highlights the need for methodological optimization and standardization.

Within the general framework described above, there was substantial variation in the details of methods for zoospore release. This variation may be due to the fact that different taxa may have different cues for release. Some may involve desiccation time (e.g., intertidal species such as *Postelsia*), or require specific light conditions for periods of photosynthesis prior to release (see review by Pearson & Serrão, 2006 for fucoids). However, there is scant information in the literature about the natural reproductive cues for different species to compare in situ natural zoospore release to lab release cues and protocols. Moreover, the variation in methodologies could be partly driven by the fact that the majority of taxa were studied in only one or a few temperate ecoregions, reflecting both the concentration of marine laboratories and researcher interests as well as the dominance and diversity of kelps in these regions (Bolton, 2010). Only a few taxa were studied across the full or major extent of their geographical range, but even in these cases there was no methodological standardization. For example, the genus *Laminaria* has been investigated across most of its geographical range, including studies from Northern California (Lewis, Green, & Afzal, 2013), Southern California Bight (Amsler & Neushul, 1991; Graham, 1999), North Sea (Bartsch, Vogt, Pehlke, & Hanelt, 2013), Celtic Seas (Han & Kain, 1992), South European Atlantic Shelf (Izquierdo, Pérez‐Ruzafa, & Gallardo, 2002), Southern Norway (Kain & Jones, 1969), and the Yellow Sea (Li, Zhou, Liu, & Wu, 1999). However, despite the abundance of studies on this genus, the methods applied to release zoospores still differed substantially across these studies. Amsler and Neushul (1991), for example, wiped the reproductive tissue with cotton towels, desiccated for 1 hr and finally immersed in unenriched artificial seawater, whereas Izquierdo et al. (2002) used a combination of wiping and rinsing of the tissue with filtered seawater, desiccation overnight and immersion in Provasoli‐enriched seawater. The genus *Ecklonia* has also been studied across most of its geographical range (e.g.,) (Akita, Yamada, Ito, Kobayashi, & Fujita, 2014; Bolton & Levitt, 1985; Graham, 1999; Jennings, 1967; Mohring et al., 2014; Novaczek, 1984a), but substantial differences in the protocols used to induce zoospore release were present in these studies (cf raw data provided on Dryad; see data availability statement). This variation of methods within genera highlights both the inconsistencies in methodology as well as the apparent insensitivity of kelps to environmental conditions to cue zoospore release.

During the pretreatment phase, different methods were used to clean the reproductive tissue from fouling organisms. If harsh chemicals or medicinal treatments were used in pretreatments, for example, bleach (Morelissen, Dudley, Geange, & Phillips, 2013), iodine (Augyte, Lewis, Lin, Neefus, & Yarish, 2018; Fox & Swanson, 2007; Varela et al., 2018), chlorine (Camus & Buschmann, 2017), or ethanol (Nelson, 2005), they were most likely an attempt to prevent contamination of the zoospore cultures by other algae or bacteria. No studies evaluated how this pretreatment may have influenced zoospore viability or subsequent gametophytes, nor the effectiveness of these treatments for their intended purpose. Similarly, desiccation treatments varied greatly, with drying times ranging from less than 15 min, to more than 2 days. It is likely that desiccation is designed to apply an osmotic shock to induce zoospore expulsion and that desiccation times are somewhat arbitrary or based on practicalities (e.g., travel time from the field to the laboratory). However, excessive desiccation appears to have a negative effect on spore release and could therefore reduce the likelihood of successful germination of zoospores (Fonck, Venegas, Tala, & Edding, 1998). Again, no studies evaluated the influence different desiccation times on zoospore viability or subsequent gametophyte performance.

Immersion media during the postdesiccation phase also varied greatly among studies and may have been driven by to the need to maximize sterile cultures or optimize nutrient availability etc., however, the use of media was rarely justified. In most cases, sterile, filtered seawater was used. It is probable that in many instances choice of media was driven by availability of fresh, sterile seawater, or cost involved in making or purchasing artificial media. In some cases, the logic underlying the choice of media was related to the actual experiment performed. For example, kelp zoospores or gametophytes used for ecotoxicological tests involved a final step of submerging the tissue in test solutions over a range of copper concentrations (Brinkhuis & Chung, 1986; Chung & Brinkhuis, 1986; Contreras, Medina, Andrade, Oppliger, & Correa, 2007). In other cases, studies performed to test the effect of ocean acidification on kelp meiospore development submerged the tissue in filtered seawater with different pH treatments (Leal, Hurd, Fernández, & Roleda, 2017a, 2017b). Regardless, immersion media are likely to be of less importance than other factors (such as temperature, desiccation time) in determining subsequent zoospore viability and performance.

Although seawater temperature plays a crucial role in the reproduction of Laminariales (Lüning, 1980), desiccation/immersion temperatures might not actually be representative of ambient seawater temperature in the region. It is likely that spore release is mechanically driven by osmotic/hydrostatic shock and that temperature is not very important. The choice of temperature range could also be based on practicalities (e.g., desiccate and submerge at room temperature).

It was surprising that given the number of studies involving the experimental release of kelp zoospores, few studies have published any attempts to quantify the influence of methodology on the resulting zoospore concentration, zoospore viability, and gametophyte performance. The exception is Fonck et al. (1998), who tested the effects of different types of prerinse, desiccation periods, and postdesiccation temperatures on sporulation, germination, and gametophyte survival for two species *Lessonia nigrescens* and *L. trabeculata*. This study found that the type of water used during the pretreatment could affect zoospore germination and survival. It also found negative effects with long desiccation periods, and positive effects with low postdesiccation temperature on sporulation. The findings of this study demonstrated the importance of testing the handling protocols, and suggested that the relationship between zoospore release density and zoospore viability is complex, warranting further study. Although this study is one of the most thorough, only three subsequent papers cited it as rationale for their methods (Edding & Tala, 2003; Tala, Edding, & Vásquez, 2004; Véliz, Edding, Tala, & Gómez, 2006) suggesting little critical evaluation of methodology across the literature. It should also be noted that very few studies have reported on the success of zoospore release at all (i.e., resulting zoospore densities). Lack of this information precludes assessments of the relative fecundity across species and environments.

## 5 CONCLUSION AND RECOMMENDATIONS

In this review, we aimed to provide guidance on best practice for Laminarian kelp zoospore extraction and review the range of successful methodologies that have been used for different taxa. This review revealed some similarities, but more often, substantial differences in the protocols used to induce zoospore release, and these could have implications for subsequent experimental results. We recommend that researchers optimize zoospore release protocols for their species to achieve an understanding of how methodology may bias subsequent experimental results (by affecting zoospore viability) but also to facilitate comparisons among studies, regions and taxa. We also recommend that at a minimum, achieved zoospore release densities are reported to aid comparisons among studies. This review has highlighted a need to standardize practices to ensure that future studies achieve optimum zoospore release. This would strengthen and facilitate comparisons of results and zoospore release densities (fecundity) across different taxa and biogeographic regions.

## CONFLICT OF INTEREST

None declared.

## AUTHORS' CONTRIBUTION

TW and MBM conceived the idea behind this study. NAA and MBM collected the literature data. NAA analyzed the data and produced all the table and figures, and led the writing of the manuscript. All authors contributed critically to the drafts and gave final approval for publication.

## Data Availability

All data included in this review are available from the Dryad Digital Repository: https://doi.org/10.5061/dryad.0kh1f8j. The data table available through this link contains a comprehensive list of the journal articles examining the microscopic stages on Laminariales, displaying the full description of the species studied; geographic location (GPS position) and ecoregion; collection date; sea surface temperature; details concerning the protocol used to induce zoospore release; aim of the research; and the reference.
